# The Common Costimulatory and Coinhibitory Signaling Molecules in Head and Neck Squamous Cell Carcinoma

**DOI:** 10.3389/fimmu.2019.02457

**Published:** 2019-10-23

**Authors:** Peng Liao, Haofan Wang, Ya-ling Tang, Ya-Jie Tang, Xin-hua Liang

**Affiliations:** ^1^State Key Laboratory of Oral Diseases, National Clinical Research Center for Oral Diseases, Department of Oral and Maxillofacial Surgery, West China Hospital of Stomatology, Sichuan University, Chengdu, China; ^2^State Key Laboratory of Microbial Technology, Shandong University, Qingdao, China

**Keywords:** HNSCC, costimulatory signaling molecules, coinhibitory signaling molecules, tumor immunity, immunotherapy

## Abstract

Head and neck squamous cell carcinomas (HNSCCs) are closely linked with immunosuppression, accompanied by complex immune cell functional activities. The abnormal competition between costimulatory and coinhibitory signal molecules plays an important role in the malignant progression of HNSCC. This review will summarize the features of costimulatory molecules (including CD137, OX40 as well as CD40) and coinhibitory molecules (including CTLA-4, PD-1, LAG3, and TIM3), analyze the underlying mechanism behind these molecules' regulation of the progression of HNSCC, and introduce the clinic application. Vaccines, such as those targeting STING while working synergistically with monoclonal antibodies, are also discussed. A deep understanding of the tumor immune landscape will help find new and improved tumor immunotherapy for HNSCC.

## Introduction

The head and neck squamous cell carcinoma (HNSCC) is the sixth most common systemic malignant tumor (1, 2). Its occurrence is closely related to exposure to tobacco, alcohol (3) and HPV infection (4). The clinical outcomes of HNSCC are always frustrating and the 5-year survival rate of early HNSCC is only 40–60%. For patients with local recurrence or/and distant metastasis, the median survival time after palliative chemotherapy is 6–9 months, while only 3–6 months for chemotherapy tolerance (2). Thus, the question of how to improve the poor prognosis of HNSCC has attracted great attention, and there is a need for further study into the molecular mechanisms of tumor growth and metastasis.

Previous studies have demonstrated that almost all types of tumors, including HNSCC, have the ability to evade the immune surveillance and clearance, resulting in tumor growth and metastasis. In an immunosuppressive tumor microenvironment (TME), there are several suppressive cytokines and chemokines, such as IL-10, TGF-β, VEGF, and PGE2, as well as negative regulatory cells, including myeloid-derived suppressor cell (MDSC), regulatory T cell (Treg) and so on. In this negative TME, the function of some effector immune cells, such as T cells and NK cells, are suppressed in various direct or indirect ways, resulting in tumor growth and metastasis (5). One such way is aimed at effector cell activation and involves the costimulatory and coinhibitory signal molecules on the surface of the immune cells.

**Figure 1:**
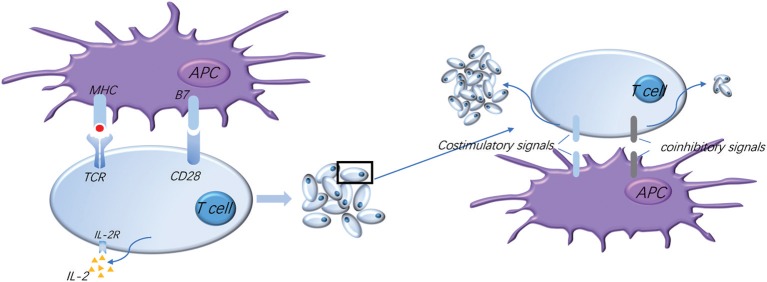
Normal activation of T cells requires costimulatory and coinhibitory signal molecules. The activation of T cells means firstly that T cells recognize the antigen presented by APC, and secondly that the costimulatory molecule B7 on the APC surface binds to the CD28 presenting on the surface of T cells to provide the second signal for the activation of T cells, which makes T cells secrete IL-2 and express high levels of IL-2R, thus promoting the proliferation and activation of T cells. Meanwhile, a variety of costimulatory and coinhibitory molecules are expressed successively on the surface of T cells: costimulatory signals further promote T cell proliferation and coinhibitory signals limit T cell activation and proliferation, which regulate T cell moderate activation accurately, effectively start the immune response, and prevent excessive immune damage to the tissue.

Costimulatory molecules as a second signal for T cell activation could promote the secretion of many cytokines and the expression of their corresponding receptor molecules, which participate in the activation and proliferation of T cells as well as the induction of T cells to differentiate them into different functional subgroups (6–8). For example, CD28 on T cells binding with B7 on an antigen-presenting cell (APC) surface exert their immune function. On the other hand, coinhibitory molecules, such as CTLA-4 and PD-1, expressed on the surface of activated T cells could play the role of the inhibitor of T cells function via a suppression signal. Therefore, the interaction between the costimulatory and coinhibitory signal molecules directly affects the function of immune cells (9, 10). And the same signal molecules on the surface of different immune cells or tumor cells play different functions, leading to a more complex TME. This review will describe the common costimulatory and coinhibitory signal molecules in HNSCC and analyze the underlying mechanism of these molecules in regulating the malignant progression of HNSCC, and it will also introduce the clinic application of mAbs for costimulatory and coinhibitory signal molecules (Figure 1).

## Costimulatory Signal Molecules

Costimulatory signals offer the requisite second signal for immune cell activation and proliferation. Most activated T cells could play an anti-tumor role in inhibiting tumor growth (11). However, the activation of regulatory T cell (Tregs) could negatively regulate T cell function and promote tumor growth in most types of cancer. Many costimulatory signaling molecules on the surface of immune cells, including CD137, OX40 and CD40, have now been found to play a vital role in HNSCC development (12–14).

## CD137

CD137 is a member of the tumor necrosis factor receptor (TNFR) families and has been regarded as a costimulatory signal receptor (12). CD137 is expressed on the surface of activated T cells, DCs and NK cells (15–17). CD137, activated by its ligand CD137L, conveys polyubiquitination-mediated signals via TNF receptor associated factor 2 and activates the NF-κB pathway that inhibits apoptosis, as well as enhances proliferation and effector functions of T cell and NK cell (18, 19). Activated CD137 could enhance the antibody dependent cell-mediated cytotoxicity (ADCC) effect through the use of NK cells (20), promote the differentiation of effector T cells, inhibit the function of Tregs and facilitate T cells to play an anti-tumor function (21). When NK cells encounter tumor cells, the expression of CD137 on NK cells increases significantly (22), helping NK cell clean tumor cells by ADCC. Besides, DC cells activated with CD137 could not only secrete IL-2 and IL-6 to promote the proliferation of T cells (23) but also activate cytotoxic lymphocytes (CTLs) and promote the secretion of IFN, hereby enhancing the anti-tumor effect (24). However, previous experiments indicated that soluble CD137 (sCD137), secreted by tumor cells in a low oxygen environment, blocked its membrane-CD137 costimulatory function, resulting in tumor escape (25). How tumor cells increase sCD137 secretion in hypoxic conditions remains unclear.

Based on the physiological effects of CD137 against tumors, Lucido et al. found that agonists for CD137 in the HPV (+) HNSCC mouse model had a synergistic effect on inhibiting tumor growth compared to traditional single radiotherapy and chemotherapy. Meanwhile, CD137L on the surface of tumor cells could also improve the efficacy of chemotherapy/radiation through the CD137/CD137L signal axis (26). Srivastava et al. showed that the use of CD137 agonists in HNSCC patients promoted the maturation of DC cells induced by cetuximab, and it also enhanced the cross-presentation function of NK cells and DC cells to HNSCC antigens. Besides, urelumab, agonistic mAbs (monoclonal antibodies) of CD137, helped inhibit the apoptosis of NK cells, playing an anti-tumor effect (27).

Two agonistic mAbs of CD137, urelumab (BMS-663513) and PF-05082566, have been developed for clinical use. Urelumab alone has a very severe hepatotoxicity and its exact mechanism is still unclear (28, 29). On the other hand, CD137 agonist mAbs could enhance other mAbs' efficacy in HNSCC patients, such as cetuximab (27). Thus, it may have an enhanced anti-tumor effect and could weaken hepatotoxicity through reducing anti-CD137 mAbs' dosage when combined with other mAbs.

## OX40 (CD134)

OX40, one of the tumor necrosis factor receptors, is mainly expressed on the surface of activated T cells, especially on CD4+ T cells (30), while the expression on CD8+T cells is low (31). What's more, OX40 is highly expressed on intratumoral T cells, particularly the FoxP3+ regulatory T-cell (Treg) lineage (32, 33). In general, the ligand of OX40 (OX40L) on the surface of DCs or MCs binds directly to OX40. Besides, bone marrow mononuclear cells (BMMCs) and some DCs could secrete exosomes containing OX40L, which remotely regulate T cell differentiation (34, 35).

OX40 activation could augment the downstream signaling of TCR mainly through the PI3-K/PKB pathway, accounting for T cell division, survival and cytokine production. Meanwhile, OX40 activated in conjunction with TCR signaling could increase calcium influx, promote nuclear factor of activated T cells (NFAT) activation and enhance several cytokines secretion, such as IL-2, IL-4, IL-5, and IFN-γ. These cytokines help promote the proliferation and differentiation of immune cells and exert anti-tumor effects. Therefore, the signaling downstream of OX40 can augment proliferation, suppress apoptosis and induce greater cytokine responses from T cells (36).

An experiment on skin squamous carcinomas *in vitro* observed that there were more OX40 + Tregs in tumor tissues than in peripheral tissues, which could inhibit the function of effector T cells and the secretion of IFN-γ. Stimulated OX40 was found to not only obviously suppress the inhibition conducted by Tregs but also reduce the number of Tregs in tumor microenvironments by activating FccRs, finally inhibiting tumor growth (32–36). However, some studies showed that OX40-stimulated Tregs by agonist mAbs retained suppressive qualities, and Tregs function had not intrinsically been impaired. The expression of IFN-γ, TNF-α, and granzyme B, which had potent anti-tumor effects, was increased significantly, and this may provide another explanation for the mechanism of OX40 (37).

OX40 could be expressed on the surface of T cells in HNSCC patients (38). Recent studies have found that the expression of OX40 on CD4(+) T cell surfaces in HNSCC patients was lower than in healthy people. Compared to patients with early tumors, the level of OX40 expressed on the CD4+ T cell surface was significantly decreased in patients with advanced tumors (39). In HNSCC, the low expression of OX40L could not help secrete adequate cytokines with anti-tumor effects (40).

A series of pre-clinical experiments have shown that anti-OX40 dose-tolerant mAb could enhance the humoral and cellular immunity of cancer patients by amplifying the effector T cells and inhibiting the function of Tregs (41, 42). In a mouse ovarian tumor, the combined application of anti-PD-1/OX40 mAb had greatly improved the anti-tumor effect (43). Besides, Gough, et al. showed that, in tumor animal models, the overall survival could be effectively improved from 50% to 100% by combining anti-OX40 therapies after complete surgery or radiotherapy (44). It indicated that OX-40 mAbs could play a synergistic role with traditional treatment (45), which provided a new promising combination treatment for HNSCC patients.

## CD40

CD40 is a costimulatory receptor molecule on the surface of APCs (DCs), monocytes and tumor cells. CD154, the ligand of CD40, is generally expressed on the surface of T cells and some innate immune cells, such as activated DCs and NK cells (46). Circulating sCD40L was higher in tumor patients, which may have a predictive role and could be an ambiguous therapeutic target (47). Binding with its ligand CD154, CD40 without enzymatic activity in the cytoplasmic domain recruits and interacts with TNF-receptor-associated factors (TRAFs), promoting the activation of the NF-κB signaling to maintain homeostasis and immunogenic pathogenic processes (48, 49). The activation of the CD40/CD154 axis results in the secretion of cytokine, transformation of immunoglobulin gene, prevention of B-cell apoptosis, increased expression of costimulatory molecules such as CD80 and CD86, formation of germinal center, production of high-affinity antibodies and formation of B memory cells (50). Furthermore, a combination of CD40/CD154 could promote antigen presentation, help effector T cells exert their role, activate mononuclear cells and down-regulate the expression of inhibitory molecules, such as PD-1 (15).

Stimulated CD40 could play a direct role in killing tumor cells (51). CD40 agonists promoted the secretion of lL-12 and reduced the expression of PD-1 on the surface of CD8+ T cells (52). Besides, anti-CD40 mAb treatment reversed phenotypic T cell exhaustion and increased the sensitivity of mAbs against anti-PD1 refractory tumors (53). In mouse tumor models, high expression of CD40/CD154 had an anti-tumor effect, and a low level of CD40/CD154 was shown to promote tumor growth. A possible explanation for this was that the former was related to IL-12, while the latter was associated with IL-10 (54–56).

As for HNSCC patients with tumor high stage, the expression of CD40 on APCs as well as tumor cells decreased, and the same applies the level of CD154 on T cells, while soluble CD40 increased in body fluids, representing a state of reduced immunity. During the whole process, the proportion of IL-12 did not change much while the content of IL-10 increased, showing an overall favorable environment for tumor growth (57). Moreover, the activation of CD40 was beneficial to the secretion of VEGF, which promoted the formation of tumor blood vessels and the growth of tumors (58).

In a study of phase III and IV of esophageal squamous cell carcinomas, the survival rate of CD40+ tumor patients was significantly lower compared with CD40– tumor patients. Besides, CD40+ tumor patients performed poorer in terms of pathological stage, distant metastasis and clinical prognosis (59). CD40+ tumor cells interacting with CD154+ activated T cells promoted the secretion of TGF and the differentiation of Th17, which contributed to the proliferation of tumor cells. Activated by CD154 or IFN-γ, the CD40 pathway in tumor cells induced the production of IL-6, promoting the progression of a variety of tumors (59, 60). However, several studies reported that stimulated CD40 may help protect bladder cancer cells from apoptosis. However, the low expression of CD40 in HNSCC was not associated with tumor cell growth. Therefore, the relationship between the expression of CD40 and tumor growth may be related to tumor types (61, 62).

So far, CD40 agonist antibodies (SGN-40, CP-870,893) are being tested in early clinical trials either alone or in combination with mAbs for lymphoma and solid tumors in humans (63). However, considering the side-effect of CD40 agonists, such as potential toxicity due to autoimmune reactions, severe cytokine release syndrome (CRS), hyper-immune stimulation syndrome leading to AICD, thromboembolic disease and tumor proliferation or angiogenesis, the use of CD40 agonist antibodies is still limited and need much more study to verify their availability.

## Coinhibitory Signaling Molecules

The surface of immune cells will also express coinhibitory molecules such as CTLA-4, PD-1, LAG3, TIM, and KIR. Modest activation of coinhibitory molecules under normal conditions coordinates the immune response and avoids excessive immunity injury. However, when the suppressive signals overexpress in the TME, the function of T cells could be inhibited, and tumor cells could get the chance to immune escape (64–70).

## CTLA-4

Stimulated by antigens or B7, CTLA-4 is expressed mainly on the surface of T cells, especially Tregs (71, 72). In a normal immune response, CTLA-4 can combine its ligand B7 with stronger affinity than CD28 does (64), resulting in two different mechanisms for T cell suppression, the rapid inhibition of T cell activation and the induction anergy of T cells. The first mechanism depends on inhibiting Akt directly by activating phosphatase PP2A, and the latter aims to replace CD28 binding with B7 (73, 74). sCTLA-4(soluble CTLA-4), mostly derived from Tregs, could play the same immunosuppression role by binding with B7 on APCs, like membrane-CTLA-4, to inhibit T-cell response. The blocking of sCTLA-4 also activated the proliferation of CD8+ and CD4+ T cells and promoted the secretion of cytokines (75).

In HNSCC patients, the expression of CTLA-4 in tumor-infiltrated lymphocytes was significantly higher than that in peripheral lymphocytes (76). In laryngeal squamous cell carcinoma, CD8+ lymphocytes showed higher expression of CTLA-4 (77). Besides, HNSCC, esophageal squamous cell carcinoma and nasopharyngeal cancer (NPC) patients with a higher expression of CTLA-4 had a worse prognosis to those with lower CTLA-4 level (78–80). It may suggest that anti-CTLA-4 mAbs could be a promising therapeutic target for cancers.

In HNSCC, CTLA-4 on Tregs had a stronger inhibitory effect on the proliferation of CD4+T cells compared with cyclic Tregs (81). The expression of CTLA-4 on CD4 (+) FOXP3 (+) Tregs in the circulation and TME increased in HNSCC patients treated with cetuximab. FOXP3(+) CTLA4(+) suppressor cells might suppress the activation of NK cells in oral squamous cell carcinomas (71). Furthermore, by using an anti-CTLA-4 antibody (ipilimumab), the inhibition capacity of Tregs to NK cells was found to be weakened (81, 82). However, the exact inhibitory mechanism between Tregs and CTLA-4 is still unclear. Some scholars believe that Tregs could achieve immunosuppression through CTLA-4 (63, 83), while other figures do not support this view (84). Currently, the preferred school of thought is that both of them play independent inhibitory roles in tumor immunity, but the inhibitory effects are overlapped (85).

Some CTLA4-blocking mAbs, such as ipilimumab and tremelimumab, are under study for further clinical importance. Ipilimumab has now been approved for the treatment of advanced melanoma by the Food and Drug Administration of America (FDA). Ipilimumab has shown astonishingly positive effects in the treatment of a variety of malignant tumors due to its synergistic effect with chemotherapy and radiotherapy (86–88). There is a new viewpoint indicating that anti-CTLA-4 antibodies induce tumor recession by the selective depletion of Tregs in tumors rather than the blocking of B7-CTLA-4 interaction in lymphoid organs (89).

## PD-1/PD-L1

PD-1 expresses on the surface of activated immune cells, such as CD4+T cells, CD8+T cells, B cells, natural killer T cells, activated monocytes, dendritic cells and macrophages (90, 91). sPD-1(soluble PD-1), interacting with PD-L1, could prevent PD-1 from binding with PD-L1 and promote effective tumor immunity, possibly resulting from decreased IL-10, TGF-β and increased IL-2 TNF-α and IFN-γ (92, 93). However, a different phenomenon had taken place: sPD-1 could inhibit T cell proliferation and IL-2 production when DCs and T cells were cocultured with sPD-1 (94).

The ligands of PD-1 are PD-L1 and PD-L2. PD-L1 expresses mainly on T cells, B cells, DCs and macrophages (95), while on some tumors surface, such as glioblastoma multiforme, NSCLC and some hematologic malignancies (96, 97). PD-L1 mRNA and proteins were up-regulated by the effect of IFN-γ, IL-4, IL-10, growth cell stem factors, LPS and VEGF (96, 98), which indicated that PD-L1 overexpression may be accompanied by immune inhibition in TME. On the other hand, the expression of PD-L1 on tumor cells could also be increased by activating intracellular signaling pathways, such as IFN-γ/JAK2/IFN, ALK/STAT3, PI3K and MEK/ERK/STAT1 (99, 100). Hypoxia-inducible factor-1 (HIF-1α) is an important factor for making tumor cells over-express PD-L1 (101). Another important source of PD-L1 is tumor exosomes, which can suppress the draining lymph node activation, inhibit IFN-γ secretion, promote immune escape and facilitate tumor growth (102, 103). sPD-L1 binding with membrane-PD-1 could also exert a wide range of inhibitory effects through the blood and lymphatic circulation (104).

Phosphorylation of the tyrosine residues in the ITIM and ITSM motifs in the cytoplasmic tail of PD-1 recruits SHP-1 and SHP-2, which, in turn, dephosphorylate proximal signaling molecules downstream of the TCR and CD28 and inhibit the activation of the PI3K/Akt and the Ras/MEK/Erk pathway (105). Hence, T cell proliferation activity, cytokine secretion capacity and cytotoxic effects are weakened, and tumor cells finally get immune tolerance (67, 68).

In HNSCC patients, PD-1 on the surface of tumor infiltrating CD8+ T cells had a higher expression, which resulted in impaired function of PD-1(+) CD8(+) T cells and facilitated tumor growth (106). In the HNSCC microenvironment, the percentage of tumor cells expressing PD-L1 was about 50–60% (107, 108). In a survey of 74 cases of primary HNSCC, Roper et al. suggested that the expression of PD-L1 was higher on tumor cells and TILs, while individual higher expression of PD - L1 (>5%) on primary tumor cells, primary TILs, and metastatic TILs was associated with longer diseases-free survival (109). Previous studies have found that higher expression of PD-L1 in gastric, breast, renal and pancreatic cancer led to poorer prognosis (110–113). However, in metastatic melanoma, Merkel cell carcinoma, HPV-associated HNSCC, mismatch-repair-proficient colorectal cancer, NSCLC and small cell lung cancer, higher expression of PD-L1 indicated a better prognosis (68, 114–118). The possible explanation may be that the expression of PD-L1 on the latter tumor cells could be induced by IFN-γ in a local inflammatory tumor microenvironment (114). In HNSCC, epithelial-mesenchymal transformation (EMT) could independently up-regulate the expression of PD-L1 on tumor cells. Compared with EMT without PD-L1 expression, the prognosis of patients with EMT-related PD-L1 expression was poorer (107). In HNSCC patients, PD-L1 levels on exosomes were associated with disease progression. The emergence of circulating PD-L1+ exosomes may be a useful metric for disease and immune activity in these patients (119).

According to the immunosuppressive function of PD-1/PD-L1, more and more blocking monoclonal antibodies have been studied and applied in clinical practice. The phase I, II, and III clinical studies have all showed that, in recurrent or metastatic HNSCC, pembrolizumab demonstrated clinically meaningful anti-tumor activity and took on a favorable safety profile (120–122).

## LAG3 (Lymphocyte Activation Gene-3)

LAG3, an inhibitory checkpoint receptor, expresses on the activated CD4+T cells (69), CD8+T cells and a subset of natural killer (NK) cells (123). For CD4+T cells, LAG3 binds to MHC II molecules with an affinity higher than CD40, while most of the molecular mechanisms remain unclear (124). For CD8+T cells and NK cells, the ligand of LAG3 is LSECtin (125). Binding to LAG3 expressing on CD8+T cells and NK cells, tumor cells could get the capacity to escape immune clearance. Besides, Tr1 cells could be identified in both humans and mice by the expression of LAG3 together with CD49b (126). But it has not been confirmed whether LAG3 is necessary for the immunosuppressive function of Tr1 cells.

In HNSCC, the increased expression of LAG3 in TILs was related to higher pathological grades, larger tumor size and positive lymph node status. However, this expression had nothing to do with several risk factors such as HPV infection. For patients with recurrent and distant metastatic HNSCC, the LAG3 level in TILs was up-regulated (127). In an immunocompetent HNSCC mouse model, Deng et al. revealed that blocking LAG-3 could suppress tumor development, potentiate antitumor response of CD8+ T cells and reduce the population of immunosuppressive cells (128). mAbs targeting LAG3 could inhibit the interaction between LAG3 and MHC-II and induce IL-2 production in a T cell assay (129).

## TIM-3 (T Cell Immunoglobulin and Mucin-Domain Containing-3)

TIM-3 is a coinhibitory receptor on IFN-γ-producing T cells, FoxP3+ Tregs and innate immune cells and suppress immune responses by interacting with TIM-3 ligand (130). Galectin-9 has the highest affinity for TIM-3. The interaction between Galectin-9 and TIM-3 triggers cell death in effector Th1 cells, dampening tissue inflammation and inhibiting autoimmune disease EAE (131). Moreover, Galectin-9 also induces cell death in Tim-3 + CD8 + TIL in colon cancer (132). Another important ligand of TIM-3 is carcinoembryonic antigen cell adhesion molecule 1 (Ceacam-1). TIM-3 was activated by the action of Ceacam-1, resulting in a weakened interaction between TIM-3 and BAT3 (an inhibitory molecule downstream of TIM3) in T cells in TIM-3 transgenic mice (133). Galectin-9 and Ceacam-1 are combined in different sections of TIM3 IgV domain (133, 134). The two ligands may therefore play a synergistic role in regulating TIM3 signals (135, 136).

In TME, tumor infiltration DCs showed higher expression of TIM-3 than normal tissue. Binding to HMGB1, TIM-3 could block the transport of nucleic acids into endosomes, suppressing pattern-recognition receptor-mediated innate immune responses to tumor-derived nucleic acids (137). TIM-3 could activate the NF-κB signaling pathway to promote tumor cell metastasis (137). In patients or animal tumor models with chronic HIV infection, the expression of TIM-3 on T cells was significantly high (138, 139). On CD8+TILs, TIM-3 often expressed together with PD-1. Besides, their co-expression had a more potent capacity to exhaust T cells compared with PD-1 alone (140–143). In advanced melanomas and NSCLCs, about 1/3 of CD8+TILs expressed TIM-3, which, co-expressed with PD-1, caused defects in the proliferation of T cells and production of effector cytokines (144, 145).

**Figure 2:**
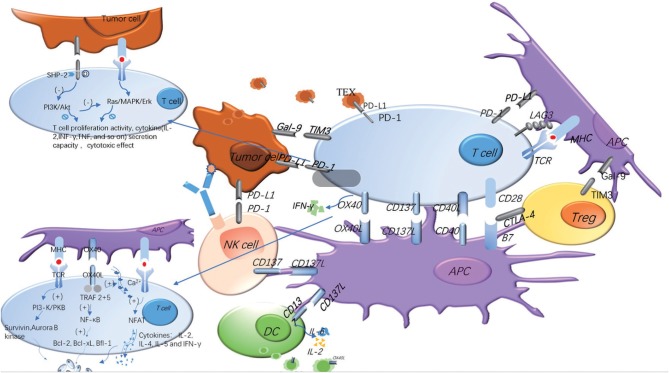
Costimulatory and coinhibitory signal molecules can be expressed on different cell surfaces and play different roles. In the process of T cells' immune response to tumors, costimulatory and coinhibitory signal molecules regulate the immune response in opposite ways. The same signal molecules may exist on the different cell surface, and the same cell surface may express many different signal molecules. The adding of TEX and soluble molecules makes the regulation of the whole immune response more complex. The blue represents the costimulatory molecules, and the gray represents the coinhibitory molecules. The signaling pathways of OX40 and PD-1 are explained in the figure.

In HNSCC patients, TIM-3+ Tregs are functionally and phenotypically distinct with TILs and are highly effective in inhibiting T cell proliferation. IFN-γ induced by anti-PD-1 immunotherapy may be beneficial to reverse TIM-3+ Tregs suppression (146). In the HNSCC mouse model, the expression of TIM-3, the percent of Tregs and CD206 + macrophages were increased, while the amount of effector T cells (CD4+, CD8+ T cells) was decreased. However, blockade of TIM-3 induced a decrease of Tregs and promoted IFN-γ production on CD8+ T cells (147). The use of anti-TIM3 antibodies could not only reduce the expression of TIM-3 on the surface of T cells, but also decrease the number of MDSCs, inhibiting tumor growth (148). Moreover, the treatment of anti-TIM-3 monoclonal antibodies could restore the function of T cells to inhibit tumor growth (149). However, some of the data indicated that TIM-3 could function as a co-stimulatory receptor to enhance CTLs and other immune cell responses, which indicated TIM-3 might play a more complex role in regulating anti-tumor responses (150–152), and much work should be done in this area (Figure 2).

## Immunotherapy Strategies of HNSCC

HNSCC is in a status of immune suppression, relating not only to the abnormal competition between costimulatory and coinhibitory signal molecules, but also a general lower immunogenicity. What's worse, about 85% of HNSCC patients are found to be resistant to immune checkpoint receptor (ICR) blockades (153). Thus, we need an effective strategy to augment the immunogenicity and inject the T cells with a “cardiac stimulant” for their anti-tumor function. Tumor specific T cell response could be induced by three classes of antigens: antigens from viral proteins (e.g., HPV), somatic mutations and those encoded by cancer-germline genes (153); vaccines may be appropriate.

In HPV-related HNSCC, vaccines containing long HPV peptides have been regarded as a new treatment to enhance tumor immunogenicity. Recently, Dharmaraj et al. produced a new type of cancer vaccine system with mesoporous silica rods (MSR), which could provide virus antigens, recruit DC cells to facilitate their maturation and transfer DCs to draining lymph nodes to promote T cell maturation (154). It suggested that the combination of targeted vaccines and an appropriate controlled-release system could produce a better synergistic immune effect.

Another effective vaccine is through the stimulation of interferon genes (STING). STING could activate STING-TBK1-IRF3 signaling pathways and secret INF-I, which plays an anti-tumor role by promoting the maturation and migration of DCs, enhancing cytotoxic T lymphocyte- or NK cell-mediated cytotoxicity effects and protecting effector cells from apoptosis (155). In humans, STING could express in the basal layer of normal skin, while STING ligands are an effective therapy for premalignant and malignant disease (156). However, SOX2 enhances the degradation of STING in an autophagy dependent manner, thereby blocking IFN-I activation. This immunosuppression state could be mitigated with a STING-inducing nanosatellite vaccine (containing a cGAMP and HPV16 E6/E7 peptide), which promotes APC maturation and enlarges tumor-specific CTLs to inhibit the immune escape of HNSCC.

Notably, the use of STING vaccines greatly improved the response of ICR-resistant HNSCC to the immune checkpoint blockade (ICB) treatment (157, 158). In the pancreatic cancer model, STING could be stimulated by the tumor antigen released after radiotherapy, or the artificial agonist that blocks M2 macrophage differentiation and decreases IL-10 secretion, and control local and distant tumors (159). Moreover, the Ataxia Telangiectasia Mutated (ATM)-inhibition could directly activate TBK1 and enhanced radiation-induced T1IFN reporter activity (160). They are all synergistic with checkpoint blockade therapy. What's more, the local delivery of STING agonists could also enhance the efficacy of surgical resection by serving as a platform to generate systemic immunity to treat or control metastatic diseases. However, the anti-tumor effect of STING agonists could be weakened by IL-10 (161). Hence, vaccines for STING combined with mAbs for signal molecules and traditional treatment methods (surgical, radiotherapy and chemotherapy) may produce more powerful anti-tumor effects (158).

## Conclusion

A variety of inhibitory and stimulatory receptors could co-express on tumor antigen-specific CD8+ T cells (including CD160, KLRG-1, TIM-3, 2B4, BTLA, and LAG3) (162). LAG3 and PD-1 could co-express in human ovarian tumor antigen-specific CD8+T cells, leading to T cell dysfunction. Simultaneous blocking of PD-1 and LAG3 could more effectively restore the function of effector T cells (163, 164). In some tumor models, anti-TIM3 has almost the same effect as anti-PD-1 and anti-CTLA-4. Blocking PD-1 and TIM3 simultaneously plays a stronger synergistic anti-tumor role. Similarly, antibodies against PD-L1, TIM3, or LAG3 could restore responses of HCC-derived T cells to tumor antigens, and combinations of those antibodies had additive effects (165). Thus, the combined application of multiple mAbs targeting at different signal molecules may bring about preferable outcomes.

Since HPV (+) and HPV (−) have been regarded as two distinct subtypes, immunotherapy for them could be different. Results displayed that HNSCC with a high T-cell inflamed phenotype (TCIP-H) were enriched in multiple immune checkpoints (particularly PD-L1, PD-L2, PD-1, TIM3, CEACAM1, LAG3, and CTLA4), had frequent mutations in CASP8, EP300, EPHA2, and HRAS, and frequent co-amplification of JAK2 and CD274. HNSCC tumors with a low T-cell inflamed phenotype (TCIP-L) were enriched in the WNT/β-catenin and Hedgehog signaling pathways, had frequent NSD1 mutations, EGFR, YAP1 amplifications and CDKN2A deletions. HPV (+) tumors were enriched in markers of Tregs, while HPV (–) tumors were enriched in M2 macrophages (166). For HNSCC patients, immunotherapy for a single molecule cannot achieve full efficiency; for example, only 13.3% of the HNSCC patients responded to anti -PD-1 (167). A combination of treatments for individuals including mAbs, vaccines, traditional methods as well as signal pathway blocking is needed.

In addition, tumor-derived exosomes (TEX) have received more attention and have been regarded as special immune checkpoints. TEX could express many different inhibitory molecules, including TGF-β1, PD-L1, CD73, and FasL on the membrane (168, 169). It could therefore suppress immune cells function and promote tumor growth (170, 171). A study of mice using an OSCC model showed that TEX could suppress tumor immune response by inhibiting proliferation of both CD4+ and CD8+ T cells and reducing infiltration of T cells into tumors, thereby promoting the carcinogenesis of murine oral squamous cell carcinomas (172). The expression of exosomes is closely related to tumor progression and immunosuppression, which may make it another promising biomarker of tumor development and immune suppression (173).

## Author Contributions

All authors listed have made a substantial, direct and intellectual contribution to the work, and approved it for publication.

### Conflict of Interest

The authors declare that the research was conducted in the absence of any commercial or financial relationships that could be construed as a potential conflict of interest.

## Abbreviations

APC: antigen-presenting cells
AICD: Activation-Induced Cell Death
CTL: cytotoxic lymphocyte
CTLA-4: Cytotoxic T-Lymphocyte Antigen-4
ESCC: esophageal squamous cell carcinoma
EMT: epithelial-mesenchymal transition
FDA: Food and Drug Administration of America
HPV: human papillomavirus
HNSCC: Head and neck squamous cell carcinoma
TCIP-H: high T-cell inflamed phenotype
ICB: immune checkpoint blockade
TCIP-L: low T-cell inflamed phenotype
MDSC: myeloid-derived suppressor cell
mAb: monoclonal antibody
PD-1: programmed death-1
Treg: regulatory T cell
Breg: regulatory B cell
DCreg: regulatory dendritic cell
sCD137: soluble CD137
sCTLA-4: soluble CTLA-4
sPD-1: soluble PD-1
TAM: tumor-associated macrophage
Th17: T helper 17 cell
TAN: tumor-associated neutrophil
ADCC: the antibody-dependent cell-mediated cytotoxicity
TEX: tumor-derived exosomes.
